# Adult‐onset severe acral angiokeratoma‐like pseudolymphoma: A case report and review of the literature

**DOI:** 10.1002/ski2.135

**Published:** 2022-05-25

**Authors:** Kana Terao‐Hirayama, Ryota Hayashi, Osamu Ansai, Tokiko Deguchi, Riichiro Abe

**Affiliations:** ^1^ Division of Dermatology Niigata University Graduate School of Medical and Dental Sciences Niigata Japan

## Abstract

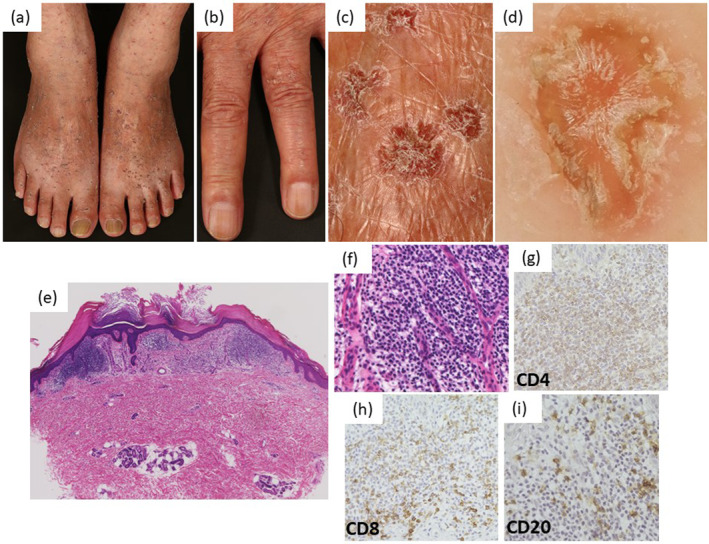

## ETHICS STATEMENT

The patient in this manuscript provided written informed consent for the publication of his case details.

Dear Editor,

Acral angiokeratoma‐like pseudolymphoma (AALP) is a rare type of pseudolymphoma that presents with dark red papules on the hands or feet.^1^ The disease was originally considered as acral pseudolymphomatous angiokeratoma of children (APACHE). However, several cases of adults with the same conditions have been reported.^1^ Herein, we report a severe case of adult‐onset AALP, and review adult onset AALP and APACHE.

**FIGURE 1:**
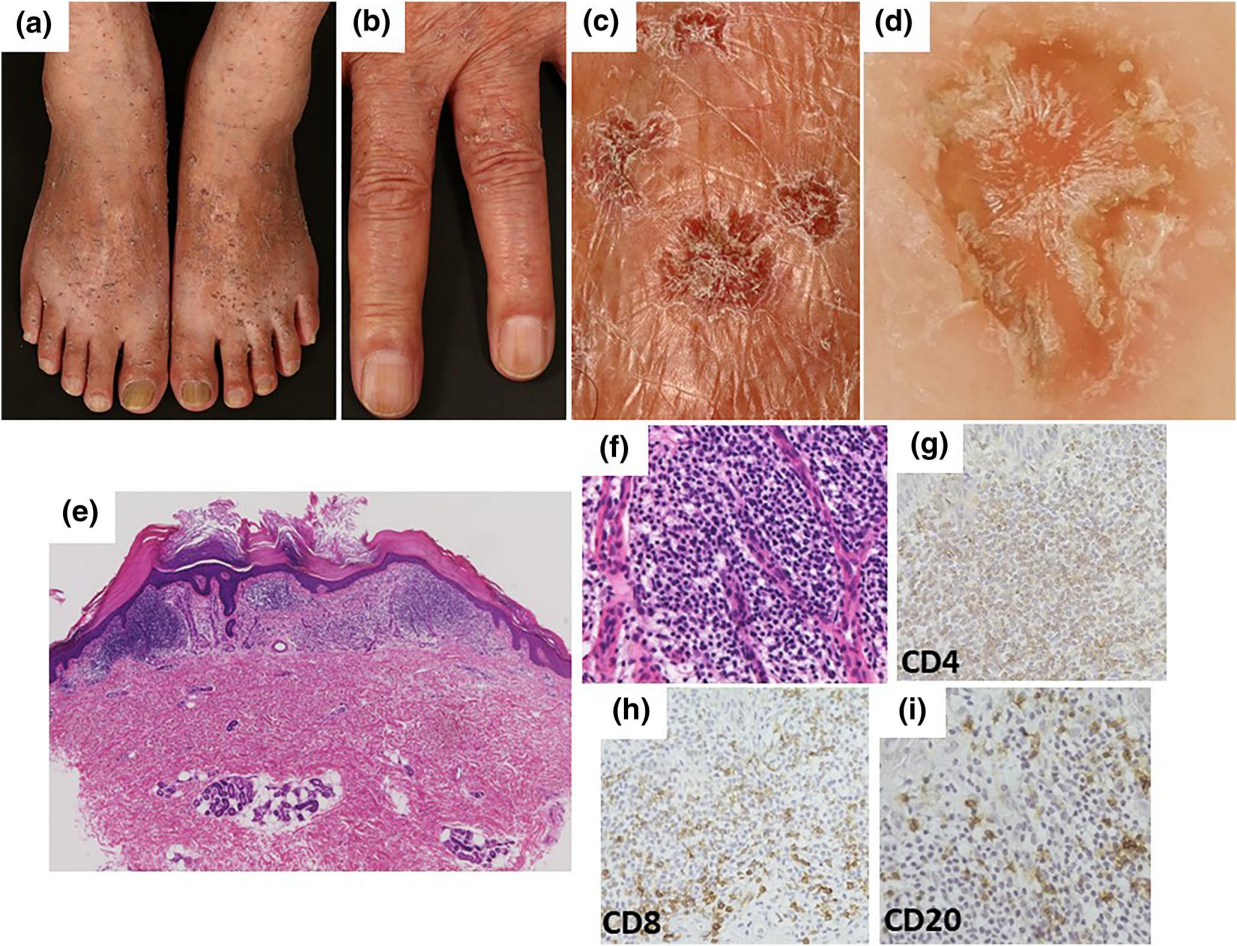
(a–c) Clinical features of the acral lesions. More than one‐hundred dark red nodules (2–7 mm in diameter) with slightly verrucous surface were observed on dorsum of the feet and fingers. (d) Dermoscopic findings showed a central yellow hyperkeratotic appearance with brown pigmentation. Red‐blue lacunas were not observed. (e–i) Histopathological findings of the patient. (e) Hyperkeratosis and many infiltrating basophilic cells in the superficial dermis were observed (Haematoxylin‐eosin staining, original magnification: ✕20). (f) Lymphocytes were densely infiltrated and capillary growth were observed. (haematoxylin‐eosin staining, original magnification: ✕200). (g–i) Immunohistochemical findings revealed the predominance of CD3 was positive cells. CD4+ cells were more numerous than CD8 positive cells. A few CD20+ cells were detected

A 74‐year‐old male with hyperkeratotic nodules and papules developed on his extremities over the past 40 years was referred to our department. Dark red nodules (2–7 mm in diameter) with slightly verrucous surfaces were observed on his extremities (Figure 1a–c). Dermoscopic findings showed a central yellow hyperkeratotic appearance with brown pigmentation (Figure 1d). Histopathological findings revealed dilated and congested vessels with a single layer of flat endothelial cells in the papillary dermis, with hyperkeratosis. The infiltrating cells in the upper dermis mainly consisted of small lymphocytes, plasma cells, and histiocytes (Figure 1e,f). Immunohistochemical analysis revealed a predominance of CD3+ cells. CD4+ cells were more abundant than CD8+ cells (Figure 1g,h). A few CD20+ cells were detected (Figure 1i). Based on these findings, an adult‐onset AALP was diagnosed.

The lesions in our case were more extensive than those in previous reports. In AALP, typical dermoscopic appearances are a combination of colours within the visible light spectrum with central whitish‐pink areas described as a rainbow pattern.^2^ In contrast, dark lacunae with dilated vascular spaces have been observed in typical angiokeratomas (AK). Although the dermoscopic findings were not consistent with AK and AALP, we initially diagnosed the patient with AK based on our clinical findings. However, histopathological findings revealed many infiltrating CD4+ T cells around the blood vessels and the diagnosis of AALP was made. Previous immunohistochemical studies showed that dermal infiltrative cells were mainly positive for T cells. Most cases featured predominant infiltration of CD4+ T cells, whereas several cases showed predominant infiltration of CD4+ and/or CD8+ T cells (Figure S1). Therefore, infiltration of lymphocytes is important for diagnosis of AALP, whereas the predominance of CD4+ and CD8+ cells appear variable in AALP.

The difference in entities between AALP and APACHE, except for the age, has not been elucidated.^1^ Moreover, whether AALP was originally caused by AK or pseudolymphoma is unknown. We assumed that the AK was originally present, and lymphocyte infiltration appeared afterwards. A previous report demonstrated that vessels of all types of AK are reportedly positive for Prox1.^3^ However, Prox1 staining was negative in our case. Therefore, in our case, we considered that pseudolymphoma had originally been excised, and thereafter, an increase in vessels and epidermal changes, such as AK occurred secondarily.

In our case, the patient reported that his father also had the same eruptions. However, as his father already died, we could not observe eruptions, and so it was not clear if his father also had AALP. A case of APACHE in adult twins has been reported^4^ and so, several cases of AALP might be involved in genetic relationships.

To the best our knowledge, 12 cases of adult‐onset APACHE and AALP are reported (Figure S1). Multiple lesions are observed in nine cases, and the eruption had occurred on fingers or toes in many cases. Most patients have unilateral eruptions and our patient's eruption were observed on his extremities, fingers, and toes (Figure S1). The treatment of APACHE and AALP is limited. Surgical excision is effective for single lesion of the disease, and topical steroids and liquid nitrogen are effective in some cases. However, these therapies have a high risk of recurrences.^5^, ^6^ Recently, CO_2_ laser and topical rapamycin were reported as therapeutic options and both therapies are effective for APACHE and AALP.^5^, ^6^ However, optimal treatment for the disease has not been cleared, and further investigation is needed. In our case, the eruption was severe than that of previous reports and he did not wish to be treated for his own eruption because the treatment of APACHE and AALP was limited.

We report a case of severe AALP and reviewed previous reports. Late‐onset symmetry hyperkeratotic lesions, such as AK should be considered as AALP.

## AUTHOR CONTRIBUTIONS

**Kana Terao‐Hirayama:** Writing‐original draft (lead). **Ryota Hayashi:** Resources (lead); Supervision (equal); Visualization (lead); Writing‐review and editing (equal). **Osamu Ansai:** Resources (supporting); Supervision (supporting). **Tokiko Deguchi:** Visualization (supporting). **Riichiro Abe:** Writing‐review and editing (equal).

## CONFLICT OF INTEREST

The authors declare no conflict of interest.

## Supporting information

Supplementary Material

## Data Availability

Research data are not shared.
