# Supplementary material for: Adult‐onset severe acral angiokeratoma‐like pseudolymphoma: A case report and review of the literature
Source: Skin Health Dis. 2022 May 25;3(1):e135. doi: 10.1002/ski2.135 (PMC9892420; doi:10.1002/ski2.135)
Supplement: Supplementary file 1 — Supplementary Material [file SKI2-3-e135-s001.docx]

**Supporting Information**

**Adult-onset severe acral angiokeratoma-like pseudolymphoma: a case report and review of the literature**

**Kana Terao-Hirayama et al.**

Figure 1. Review of adult cases of acral pseudolymphomatous angiokeratoma of children/ Acral angiokeratoma-like pseudolymphoma collected from the literature.

| **Reference** | **Age/gender** | **Duration** | **Number of lesions** | **Location** | **Immunohistochemistry** |
| --- | --- | --- | --- | --- | --- |
| Kaddu at al.^1^ | 64/F | 1 year | single | back | N.A |
| Okada et al.^2^ | 52/F | 4 years | multiple | right hand | CD4>CD8, lowCD20 |
| Okada et al.^2^ | 41/F | 1 year | multiple | right first toe | N.A |
| Hagari et al.^3^ | 73/M | 3 months | multiple | lower extremities | CD4=CD8, low CD20 |
| Ohtsuka et al.^4^ | 28/F | 6 months | multiplel | left foot and sole | CD3, CD4 positive |
| Okuyama et al.^5^ | 21/F | several years | single | right heel | CD4=CD8, low CD20 |
| Okuyama et al.^5^ | 34/F | >10 years | multiple | right thigh | CD4=CD8, low CD20 |
| Chedraoui et al.^6^ | 76/M | 5 years | multiple | extremities | CD4=CD8, low CD20 |
| Fonia et al.^7^ | 40/M | 8 years | multiple | left foot | CD4=CD8, low CD20 |
| Fonia et al.^7^ | 40/M | >20 years | multiple | N.A | N.A |
| Geller et al.^8^ | 59/F | 2 years | single | left first toe | CD4>CD8 PD-1 positive |
| Mercuri et al.^9^ | 30/F | 14 years | multiple | right toes | CD3 positive |
| Present case | 74/M | >40years | multiple | extremities, finger, toe | CD4>CD8, low CD20 |

M: Male, F: Female, N.A: not available

**REFERENCES**

1. Kaddu S, Cerroni L, Pilatti A, Soyer HP, Kerl H. Acral pseudolymphomatous angiokeratoma. A variant of the cutaneous pseudolymphomas. Am J Dermatopathol 1994;**16**(2):130-3.
2. Okada M, Funayama M, Tanita M, Kudoh K, Aiba S, Tagami H. Acral angiokeratoma-like pseudolymphoma: one adolescent and two adults. J Am Acad Dermatol 2001; **45**(6 Suppl): S209-11.
3. Hagari Y, Hagari S, Kambe N, Kawaguchi T, Nakamoto S, Mihara M. Acral pseudolymphomatous angiokeratoma of children: immunohistochemical and clonal analyses of the infiltrating cells. J Cutan Pathol 2002;**29**(5):313-8.
4. Ohtsuka T, Yamazaki S. Acral angiokeratoma-like pseudolymphoma in a 28-year-old Japanese woman. Dermatology 2003;**207**(1): 77-8.
5. Okuyama R, Masu T, Mizuashi M, Watanabe M, Tagami H, Aiba S. Pseudolymphomatous angiokeratoma: report of three cases and an immunohistological study. Clin Exp Dermatol 2009;**34**(2):161-5.
6. Chedraoui A, Malek J, Tamraz H, Zaynoun S, Kibbi AG, Ghosn S. Acral pseudolymphomatous angiokeratoma of children in an elderly man: report of a case and review of the literature. Int J Dermatol 2010; **49**(2): 184-8.
7. Fonia A, Bhatt N, Robson A, Kennedy CT. Acral pseudolymphomatous angiokeratoma of children (APACHE)-like eruption in adult identical twins. Clin Exp Dermatol 2016; **41**(7): 751-3.
8. Geller S, Markova A, Pulitzer M, Myskowski PL. Acral angiokeratoma-like pseudolymphoma in a middle-aged woman. J Cutan Pathol 2017; **44**(10): 878-81.
9. Mercuri SR, Paolino G, Bartolucci M, Rizzo N, Brianti P. Acral pseudolymphomatous angiokeratoma of children (APACHE): Dermoscopic features and successful treatment with CO 2 laser. Dermatol Ther 2018;**31**(5):e12682.
